# “The Only Place I Feel Understood”: A Mixed Methods Survey of Adolescent and Caregiver Perspectives on Social Media and Parental Controls

**DOI:** 10.1177/13591045261417801

**Published:** 2026-02-24

**Authors:** Melanie Watt Ramos, Constance M. Wiemann, Lauren A. Bretz

**Affiliations:** 1 Department of Pediatrics, Division of Adolescent Medicine and Sports Medicine, 3989 Baylor College of Medicine and Texas Children’s Hospital, Houston, TX, USA; 2 Department of Pediatrics, University of Tennessee College of Medicine/LeBonheur Children’s Hospital, TN, USA

**Keywords:** adolescent, social media, eating disorder, parental controls, mental health

## Abstract

**Purpose:**

Given adolescents’ extensive social media use and its mixed impacts, this study explores: (1) adolescent and young adult (AYA) social media habits, (2) familial factors associated with parental controls, and (3) social media use among AYA with disordered eating.

**Methods:**

AYA aged 11–20 visiting an urban academic medical center and fluent in English or Spanish were surveyed on social media use and mental health. Caregivers of minors provided consent and data on demographics and parental controls. The survey included multiple choice, yes/no, and free-text questions. Analyses included descriptive statistics, correlations, and qualitative coding.

**Results:**

Participants averaged 15.3 years; 46% spent 4+ hours on social media daily. Younger adolescents (11–14) were more likely to report high use (*p* = 0.002) and have parental controls (*p* = 0.006). Most AYA (76%) felt better after using social media. Over half of parents (54.4%) used controls. Qualitative themes included: social media’s unrecognized benefits, its dual role in time use, and its value for health information.

**Conclusion:**

AYA generally viewed social media positively, though many wished to reduce usage. Parental controls focused more on content than time. Social media supported some with eating disorders, though 30% encountered harmful content.

## Plain Language Summary

Almost all teens and young adults use social media. In the past, studies showed that young people mostly see the good effects, while adults often worry about the harmful effects. This study asked teens and their parents about how they use social media and how it makes them feel. Most teens said they had good experiences online, but many also said they wanted to spend less time on it. Most caregivers also use social media. About half of them had parental controls to help manage their child's use. Caregivers with at least a high school education and parents of kids aged 11 to 14 were more likely to use these controls. Having controls did not lower their children's time online. For teens who had eating disorders, many said social media helped them feel better, but some said it made things worse. The study shows that doctors and other adults who work with teens should talk about both the good and bad sides of social media.

## Background

Social media, defined as a digital creation platform where users can interact and share with others (American Academy of Pediatrics, 2025), is widely utilized among adolescents and young adults (AYA), with 98% having used at least one platform (Nesi, 2020) and nearly one-third reporting they are on social media “almost constantly” (Vogels et al., 2022, Madden et al., 2024). Prior research has identified both positive and negative impacts of social media use (Kerr et al., 2025). Social media exposes adolescents to cyberbullying, racist content, and hate speech related to gender identity (Nesi, 2020; Bickham et al., 2022, O’Reilly et al., 2018), but it has also been associated with increased community connection for minority youth (Selkie et al., 2020), increased cognitive and affective empathy (Valkenburg et al., 2022), and acute improvement in mood (Minich et al., 2023). With 95% of AYA having access to a smartphone (Alonzo et al., 2021), studying the intersections between social media use, parental controls, and mental health has timely impact.

One area in which social media has had mixed impact is in AYA with eating disorders. One in three girls who use TikTok (31%) and Instagram (32%) say they feel bad about their body and report body dissatisfaction (Nesi, 2020). Both high frequency and high-volume social media use have been associated with development of an eating disorder (Dane & Bhatia, 2023; Sidani et al., 2016). Social media can also provide communities that support recovery from eating disorders, though content often is not produced by medical professionals, lacks moderation and the promotion of disordered eating practices can be hidden within recovery content (Sjöström et al., 2024). Those undergoing active treatment for eating disorders have expressed a desire for parental engagement in helping manage their TikTok feeds but worry that their parents might not understand the platform well enough to provide this aid (Sjöström et al., 2024).

Though the research on positive impacts of social media use is expanding, caregivers and providers for adolescents are more likely to focus on potential harms (Mekonen et al., 2024). Caregivers worry about exposure to explicit content, oversharing personal or family information, and impact on school performance (Vogels et al., 2022). Adolescents, in contrast, emphasize the positive impacts (Mekonen et al., 2024) and report stress, body image, mood, and relationships are all better after time on social media (Carter et al., 2023). The disconnection between parent and adolescent perspectives leads to regular arguments about phone use in nearly four in ten (38%) families (Alonzo et al., 2021).

Parental controls are one method parents use to address concerns about their adolescents’ social media use. Nearly three in four parents (76%) say that managing time spent on devices is a priority (Alonzo et al., 2021), though this number varies across studies. A national survey of 13-17-year-olds reported one in three (31.8%) participants had no screen time rules and those who did more often had content rules (36.2%) rather than time limitations (12.4%) (Bickham et al., 2022). About half of parents (50%) have looked through their teen’s phone, with this occurring more often in younger adolescents (64% among 13–14-year-olds) and decreasing as they age (41% among 15–17-year-olds) (Alonzo et al., 2021). Various factors are associated with parental prioritization of screen time management. Latino (25%) and Black (24%) parents are more likely to name this as a top priority when compared to white parents (10%) (Faverio et al., 2025). In households with annual income less than $30,000, screen time management is more often a top priority (22%) compared to households with incomes >$75,000 (10%) (Alonzo et al., 2021). There are no published studies of which the authors are aware on the impact of family size and parent education on presence of parental controls.

As noted above, Latino parents are likely to name screen time management as a top concern. In the United States, the Latino population is the fastest-growing minority, expected to make up 28% of the population by 2,060 (Coral et al., 2024). This population is more likely than the non-Latino white population to seek out health information online (Rivera et al., 2025; Calvin et al., 2024) and in Hispanic adolescents, this has increased over time (Landry et al., 2015). To the authors’ knowledge, no published literature evaluates social media perspectives by Latino AYA. This study is set in a predominately Latino city (U. S. Census Bureau, 2025) and sought to better characterize the social media use of this unique population.

While evidence regarding positive impacts of social media is expanding, the negative impacts cannot be overlooked, especially in adolescents with eating disorders and other mental health diagnoses. Thus, further information regarding adolescents’ perspectives of their social media use and familial factors that are associated with presence of parental controls is needed. This will aid in developing educational resources for parents and adolescents to guide them through the AYA period. This study is set in an adolescent specialty clinic, with a third of patients being treated or evaluated for an eating disorder. This study aims to (1) survey AYA regarding their perspectives on social media use, (2) evaluate familial factors associated with parental controls; and (3) explore social media use in AYA with eating disorders.

## Methods

This study was approved by the authors’ affiliated academic Institutional Review Board for Human Subjects Research.

### Participant Selection

Patients between the ages of 10 and 24 who presented for subspecialty adolescent medicine outpatient visits at one of two clinic settings (urban and suburban locations) in a tertiary urban children’s hospital in the United States were approached for enrollment. Ultimately, recruitment yielded participants ages 11 to 20. Of clinic patients, approximately one-third are undergoing evaluation and treatment for an eating disorder with the remainder presenting for mental health management, reproductive health, weight management, or acute sports injury. Inclusion criteria were English- or Spanish-speaking caregivers, English-reading adolescent, development approximately consistent with chronologic age, and smartphone access. Patients were excluded if they did not have access to a smartphone, were presenting for a new concussion evaluation, or did not read or read English. Participants ≥18 consented for themselves. Caregivers of patients under 18 years of age provided minor consent and responded to a set of survey questions themselves. AYA were compensated $20 for survey completion. Caregivers were not compensated.

### Survey Design

The survey was created to address the study aims based on identified gaps in previously reported literature. Based on the definition of social media by the AAP (American Academy of Pediatrics, 2025) and the social media platforms referenced in existing literature, the survey contained a list of platforms commonly used by both adults and adolescents (Appendix). Respondents could add any platform not mentioned. A panel of experts (adolescent medicine, psychology, child development) evaluated the survey questions to establish content validity. It was piloted with five participants who reported the time it took to complete the survey and made suggestions about questions they would change or remove. The five pilot respondents were not included in the final data analysis. Data were collected in the form of multiple choice, single choice, Likert scale, and free text responses (Appendix). Based on branching questions, maximum number of possible questions was seven for caregivers and forty-three for adolescents. Average completion time was 4–5 minutes.

The AYA survey collected demographic characteristic data regarding age, race, ethnicity, sex assigned at birth, and gender identity. The remaining questions asked about social media use, specifically platforms used (Figure 1), the number of hours spent on social media daily, if eating disorder content is viewed, and if so, if such content is perceived as helpful or harmful. Use of social media was not required for enrollment, as there was a branching option to the survey that asked why the adolescent is not on social media. Caregivers were surveyed regarding education level, number of children, whether they use social media and which platforms (Figure 1), and parental social media controls.

**Figure 1.**
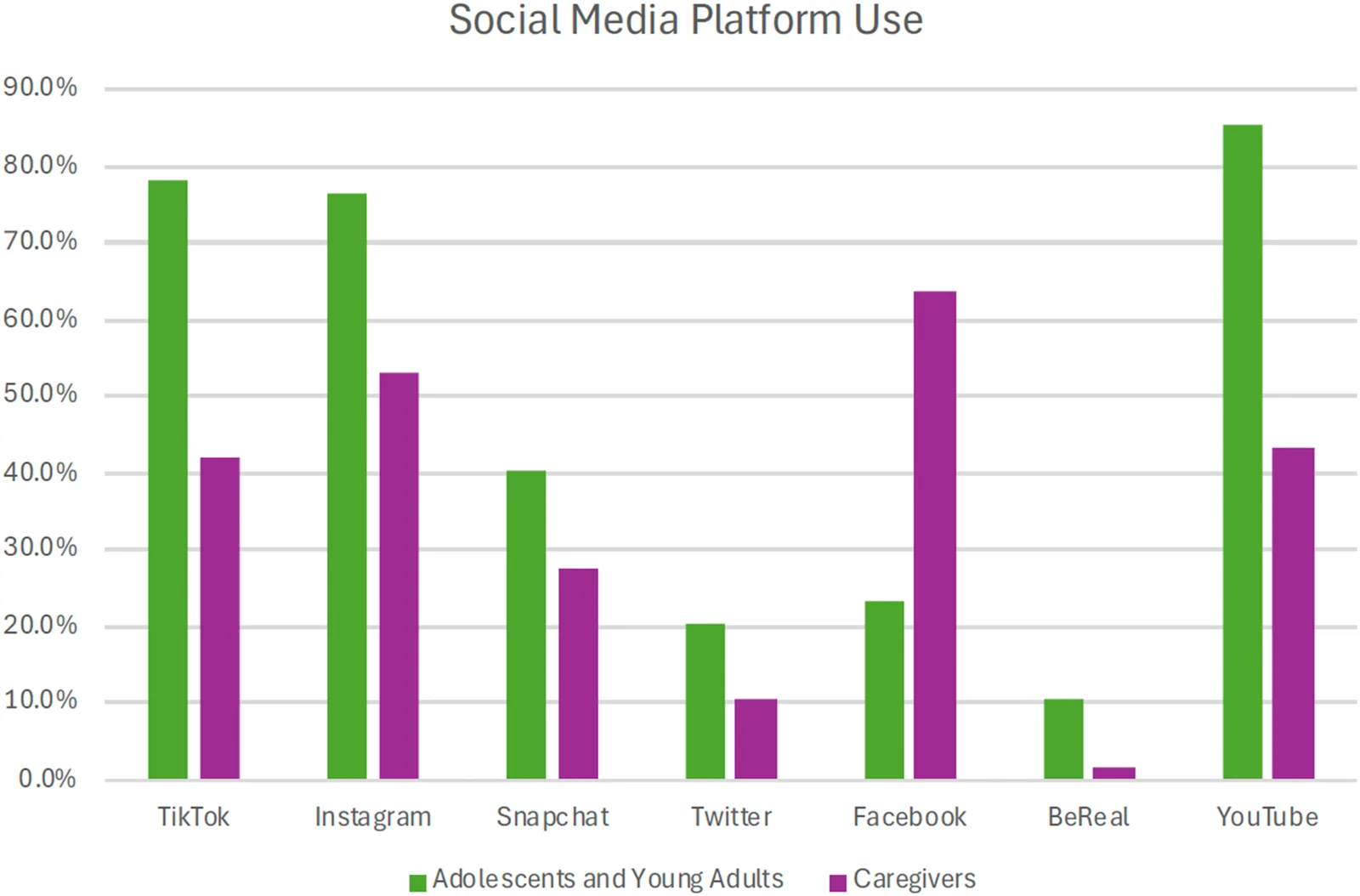
Percent of AYA and caregivers who use different social media platforms

### Data Collection

Both AYA and caregivers used a QR code to access the HIPPA-compliant REDCap (Version 15.0.26) survey. For participants under 18, the first questions were for the caregiver, with the final question confirming consent for the adolescent to participate. Caregiver questions were written in both English and Spanish. Adolescent questions were in English only, reflecting the demographic profile of the patient population.

### Statistical Analysis

Descriptive statistics (e.g., frequencies, means, and standard deviations) were used to summarize quantitative data. Quantitative data were analyzed using Chi-square and independent sample t-tests. Free-text survey questions responses were reviewed and coded by two independent reviewers (MWR and LB). Discrepant codes were discussed until consensus was reached. Codes were organized into subthemes and then combined into higher level themes.

## Results

### AYA and Caregiver Demographic Characteristics

Of the 124 AYA participants, 101 were minors whose caregivers also completed survey questions. AYA averaged 15.3 years (SD: 2.1; range: 11 – 20), were predominately white (69.4%), Hispanic (61.3%), and assigned female at birth (75.8%) (Table 1). Of all AYA participants, 52 (43%) had a history of an eating disorder. This demographic profile matches that of the clinics from which they were recruited.

**Table 1.** AYA Demographic Characteristics

Participants	(N = 124)
Average age, years	15.3 (SD: 2.1; range 11-20)
Race	
White	86 (69.4%)
Black	19 (15.3%)
Asian	4 (3.2%)
American Indian/Alaska Native	3 (2.4%)
Two or more	12 (9.7%)
Hispanic/Latino ethnicity	76 (61.3%)
Female sex assigned at birth	94 (75.8%)
Gender	
Female	88 (71%)
Male	30 (24.2%)
Non-binary	3 (2.4%)
Other	3 (2.4%)
Eating disorder diagnosis	52 (43%)

The 101 caregivers reported an average of 2.6 children (range: 1-6) and had achieved a high school education or higher (67%). The percent of caregivers with a high school education was lower than the metro area (82.3%) and the state (84.6%) (Understanding Houston, nd).

### Social Media Use and Time

Nearly all (*n* = 121, 97.6%) AYA were users of social media. Per self-report, about half of the AYA (54%) spent 1–4 hours daily on social media and half (46%) spent 4+ hours. Eleven-to fourteen-year-olds (60%) were more likely than their 15–20-year-old (32.4%) counterparts to spend 4+ hours on social media daily (*p* = 0.002, effect size 0.274). When divided into three age ranges (11–13, 14–17, and 18–20), 14–17 year-olds (33.3%) were less likely to spend 4+ hours on social media than 11-13 (62.1%) and 18-20 year-olds (55.6%) (*p* = 0.017, effect size 0.260). Presence of parental controls was not associated with time spent on social media (*p* = 0.764).

Most of the 101 caregivers reported social media use (91.3%) (Table 1). Adolescents were more likely to use YouTube (85.5%), TikTok (78.2%), and Instagram (76.6%) while caregivers were more likely to use Facebook (63.7%), Instagram (53.2%), and YouTube (43.5%) (Figure).

### Parental Controls

Slightly more than half of caregivers (54.4%) reported having parental controls in place (Table 2). Parental controls are more common among caregivers with adolescents age 11-14 (67.3%) than those with adolescents age 15-20 (41.2%) (*p* = 0.008, effect size 0.262). Caregivers with more than a high school degree (60.9%) were more likely to have parental controls in place than those with a high school degree or less (41.2%) (*p* = 0.047, effect size 0.186). Number of children was not associated with presence of parental controls (*p* = 0.98). Nine (8.7%) parents reported that they did not read or understand the language in which their child navigates the internet (Table 2).

**Table 2.** Caregiver Demographic Characteristics

Caregivers	(*N* = 103)
Average number of children	2.6 (SD: 1.2, range 1-6)
User of social media	94 (91.3%)
Parental controls	56 (54.4%)
Education level, high school or higher	69 (67%)
Does not read the language their child uses to navigate the internet	9 (8.7%)

### Eating Disorders, Mental Health, and Social Media Use

When asked about time on social media, most AYA reported that: social media was helpful or somewhat helpful (72%), they feel better or somewhat better about themselves after time on social media (76%), and social media has a positive or somewhat positive impact on mental health (75%). AYA top reasons for using social media are connection (76%), distraction (70%), and to pass time (65%). Over half (56%) of AYA participants viewed content from people with active or recovering eating disorders. Of those with a self-reported mental health diagnosis, 46% use social media to find support. While those in therapy for a mental health diagnosis spend more time on social media (35.8% spend 4+ hours daily), it was not statistically significant. Those currently taking medication for mental health (45.5%) were more likely than those not on mental health medication (14.3%) to spend 4+ hours daily on social media (*p* = 0.041, effect size 0.297).

AYA with an eating disorder and without an eating disorder did not differ in the amount of time spent on social media (*p* = 0.65). Of those with an eating disorder, 71% reported finding helpful eating disorder content.

### Free-Text Response Results

Participants were given the opportunity to provide free-text responses to questions regarding how time on social media is helpful or harmful, how they might want to change their time on devices, and what they want adults in their lives to understand regarding their time on social media. Three major themes emerged from those responses: Social media has benefits that adults do not recognize, Social media can both fill time and waste time, and AYA seek health information and support on social media. Table 3 contains a description of each theme with exemplary quotes.

**Table 3.** Themes and Subthemes of AYA Responses to Open-Ended Questions, and Exemplary Quotes

Themes/Subthemes	Description	Data Sample
Positive AYA perspective • Learning new skills • Finding social connection • Reducing stress • Feeling not alone	Participants have an overall positive perspective on social media use. They use the platforms to learn new skills, connect with peer they know in real life, reduce stress. They express that social media helps them to feel like they are not alone.	• “My time on social media lets me learn new things and helps me pass time.” (AYA #115) • “Social media has a lot of perks, and I use it to educate myself and also entertain myself [and] even to promote my businesses at times.” (AYA #121) • “It lets me know I’m not alone in recovery [from an eating disorder]” (AYA #26) • “It allows me to realize [I’m] not alone and that there are many people that suffer the same way I do and their journey while recovering, showing everybody the meaning of having a [eating disorder].” (AYA #100) • “My time on social media is mostly time for me [to] relax. [It] is kind off a way to escape reality and not worry about what’s going on around me or in the world.” (AYA #103) • “I generally use social media as a stress outlet as it helps me reduce any anxiety or anger [I] might be feeling. I Think my use of this luxury is more good than bad, especially when [I] use it as entertainment or just to have something in common to talk about with friends.” (AYA #134) • “I want adults to know that it's not all bad and there a lot of positive people on social media encouraging me and motivating me to be a better and healthier person in general.” (AYA #95)
Relationship to time • Time filler • Desire to reduce time • Self-imposed limits	Participants discussed the relationship between time and social media use. Many identify social media as a time filler, but many also note that they have a desire to reduce the amount of time they spend on social media, and some have even implemented self-imposed limits.	• “I use social media as a source of distraction when I am bored.” (AYA #28) • “That I am not harmed or discouraged [in any way] while I am on social media. [It’s] more of a tool I use when I am board and just want to be entertained.” (AYA #50) • “It’s often used as a distraction or a tool for procrastination, but more often makes me feel worse than not. Screen time also increases my anxiety because I’m worried about how it affects my eyesight.” (AYA #64) • “I wish that I used my phone less because sometimes it distracts me from homework.” (AYA #95) • “What [I] would change on the way I use my phone is by excessively just scrolling on it for hours which makes me unproductive.” (AYA #127) • “I have put my own limits in place such as 7 minutes on Instagram, and I mostly use LinkedIn and YouTube (to learn school content). Overall, I feel that I have a much better relationship with social media than many people my age.” (AYA #34) • “I have an app in place I put to lock the apps at 7:30 so I’m actually able to sleep.” (AYA #46)
Health information and support • Diet content • Health resources • Eating disorder recovery support	Social media is used as a source of health information and support. This ranges from content that encourages disordered eating or glorifies dieting to helpful health information. Those with eating disorders find social media as a source of recovery support.	• “Information that encourages dieting or outlines how to become skinny/undergrowth is exactly what got me in the hospital for an eating disorder. Reality TV shows, YouTube series, or general dieting advice are readily accessible to young, self-conscious teenage girls who don’t understand that being skinny does not necessarily equal being healthy.” (AYA #23) • “I’ve seen people brag about binge eating or not eating for long periods of time for views and likes.” (AYA #132) • “All of the content that I found related to [eating disorders] helps people stay safe and encourages to stop purging.” (AYA #54) • “I don’t really use social media because of parents but [I] mostly like watching YouTube videos about workouts, calorie counting, and Christian quotes and prayers.” (AYA #118) • “[Online creators] say similar things that my nutritionist says.” (AYA #11) • “A lot of what I see is supportive, such as challenging fear foods.” (AYA #34)

#### Positive AYA Perspective

AYA reported many benefits of their time on social media, including feeling less alone and functioning as a coping tool and way to relieve stress: “It allows me to realize I’m not alone and that there are many people that suffer the same way I do and their journey while recovering, showing everybody the meaning of having an [eating disorder],” (AYA #100) (Table 3).

#### Relationship to Time

Participants identified social media as a time filler they often turn to when bored or seeking distraction or procrastination and expressed a desire to reduce time spent on social media: “I have an app in place I put to lock the apps at 7:30 so I’m actually able to sleep” (AYA #46). (Table 3).

#### Health Information and Support

AYA reported using social media as a way to find health information that was both helpful and harmful: “I’ve seen people brag about binge eating or not eating for long periods of time for views and likes” (AYA #132) (Table 3).

## Discussion

This study sought to survey AYA regarding their perspectives on social media use, evaluate the familial factors that impact parental controls, and explore social media use among AYA with eating disorders. The main findings from this study are that social media is widely used by both AYA and caregivers, AYA view social media as positive overall, but also report viewing harmful content, and while about half of caregivers reported using parental controls, it did not impact youth time spent on social media. The overall positive perspective AYA have of their time on social media is consistent with existing literature (Bickham et al., 2022; Carter et al., 2023). However, many adolescents expressed a desire to reduce the time they spend on social media, congruent with 36% of teens reporting they spend “too much time on social media” (Vogels et al., 2022).

### Parental Controls May be Impacted by Platform Use Discrepancy

Presence of parental controls was not associated with time on social media, perhaps because parental controls are more likely to limit content the youth can see, apps they cannot use, or places they cannot use devices (Bickham et al., 2022) rather than the amount of time spent on devices. Discrepancy between platforms used by caregivers and those used by AYA also presents challenges, with caregivers potentially needing to implement structure around platforms they do not use or understand. This disconnect demonstrates a need for further resources to aid AYA and their caregivers in setting boundaries around social media.

### Younger Adolescents Spend More Time on Social Media, in Whom Brain Development May Not be Prepared for Risks of Use

In contrast to previous national polling that found that 17-year-olds spend the most time on social media (Brenan, 2024), we found significantly higher use of social media among younger adolescents, with a decrease in the 15–17-year-old age group. To the authors knowledge, no other published studies separate younger adolescents (11–14) from older adolescents (15–20) regarding time spent on social media. Theories as to the reduction in use among 15–17 year-olds include more time spent on extra-curricular activities or homework and higher likelihood of working a job while attending school as compared to their 11–13 year-old counterparts; this would be notable to explore in future research. Younger adolescents who spend large amounts of time on social media may be at higher risk for viewing harmful content or being taken advantage of by other users due to their developmental stage. This is of concern because the prefrontal cortex, responsible for executive function, and the limbic system, responsible for emotional response, do not develop at the same pace. It is not until about age 15 that the adolescent brain reaches capacity to reason potential risks and harm associated with a hypothetical situation, which is required for social media readiness. Even then, they are still likely to make poorer decisions in periods of heightened arousal or intense emotion (Arain et al., 2013). Social media creators often create emotional intensity in order to increase engagement, but that artificial emotional intensity may lead adolescents to make poorer decisions such as viewing explicit content, trying unsafe “health trends” or sharing personal or family information that should not be shared online. Parents limiting content and app usage can potentially counter the likelihood of a young adolescent encountering harm, but high-volume exposure of social media to the developing brain remains an underexplored area.

### Eating Disorder Patients Encounter Harmful Information

Time on social media was not associated with the presence of an eating disorder. However, nearly 30% of participants with eating disorders reported interacting with eating disorder content that they deemed harmful. TikTok content that promotes disordered eating behavior, often called “pro-ana,” has been found to decrease body-image satisfaction, even when time on the platform is limited (Blackburn & Hogg, 2024). This has been found in other studies looking at the intersection of TikTok use and body image, but not all (Conte et al., 2025). As discussed in the introduction, both high-frequency and high-volume use of social media have been connected to development of an eating disorder (Dane & Bhatia, 2023; Sidani et al., 2016). Adolescents increasingly use social media as a source of health information (Landry et al., 2015), with 40% of Gen Z stating that they believed health information from social media is as reliable as information from a healthcare provider (Weber Shandwick & KRC Research, 2023, Calvin et al., 2024). However, analysis of TikTok videos for a specific health condition found 44% of videos contain non-factual information (Niewijk, 2024). This underscores the need for healthcare providers and health systems to engage AYA via social media platforms to ensure evidenced-based information is shared in an easily consumable manner.

### Social Media Use in Those on Medications for Mental Health is High, Prompting Provider Screening

Use of mental health medications for an eating disorder are not routinely indicated unless there is a coexisting mental health diagnosis, including anxiety, depression, or obsessive-compulsive disorder (Bodell et al., 2012). In this study setting, where patients are primarily seen for eating disorders and a small minority are seen for primary mood and anxiety diagnoses, it is possible that patients on mental health medications may have co-occurring diagnoses. When a mood disorder co-occurs with an eating disorder, psychosocial functioning and overall prognosis are worse than in patients without a concurrent mood disorder (Coral et al., 2024; Hughes et al., 2013; Pollack & Forbush, 2013). A recent survey of nearly one thousand adolescents demonstrated that those with a mental health status described as “troubled” spent more time on social media (Blackwell et al., 2025) This knowledge, along with the finding of those on medication being more likely to spend 4+ hours on social media, suggests that providers should prioritize screening for both time and content on social media in those patients with mental health diagnoses.

### Strengths and Limitations

Limitations of this study include use of a newly created survey and sampling from a single institution, thereby limiting generalizability of study findings. In addition, although nearly all adolescents at our institution speak English, the adolescent survey was not available in Spanish; thus, the results do not generalize beyond English-speaking adolescents. Strengths include the sample size and use of qualitative and quantitative data collection, as well as a unique look at social media perspectives in the Latino population, the fastest-growing minority population in the United States.

## Conclusions

Given the ever-changing landscape of social media, it is important to understand that adolescents have an overwhelmingly positive view of their use while also noting potential harm of content and a desire to reduce the time they do spend. Findings also contribute further information on the familial and parental factors that impact parental controls. This suggests a need for further research on methods to aid adolescents in their desire to reduce time on social without increasing familial discord. Lastly, the data highlight the importance of acknowledging the positive impacts of time on social media while addressing potential harmful impacts in a developmentally appropriate manner.

## Supplemental Material

Supplemental Material - “The Only Place I Feel Understood”: A Mixed Methods Survey of Adolescent and Caregiver Perspectives on Social Media and Parental Controls Supplemental Material for “The Only Place I Feel Understood”: A Mixed Methods Survey of Adolescent and Caregiver Perspectives on Social Media and Parental Controls by Melanie Watt Ramos, Constance M. Wiemann, Lauren Bretz in Clinical Child Psychology and Psychiatry

## Data Availability

Deidentified data will be made available upon request.*
