# Supplementary material for: “The Only Place I Feel Understood”: A Mixed Methods Survey of Adolescent and Caregiver Perspectives on Social Media and Parental Controls
Source: Clin Child Psychol Psychiatry. 2026 Feb 24;31(4):1577–90. doi: 10.1177/13591045261417801 (PMC13620244; doi:10.1177/13591045261417801)
Supplement: Supplementary file 1 [file sj-pdf-1-ccp-10.1177_13591045261417801.pdf]

Appendix. Selected questions from survey.

Caregiver questions:

1. How many children do you have?
2. What is your highest level of education?
    a. 8th grade or less
    b. Some high school
    c. GED or high school diploma
    d. Some college
    e. College graduate
    f. Masters degree
    g. Doctorate degree
3. Are you a user of social media? If so, which platforms?
4. Do you put any parental controls in place for your child(ren)s social media use?
    a. Yes or No
5. Does your child navigate the internet in a language you can read and understand?

AYA Questions:

1. Age
2. Race
3. Ethnicity
4. Gender identity
5. Are you a user of social media? If so, which platforms?
    a. TikTok
    b. Instagram
    c. Snapchat
    d. Facebook
    e. BeReal
    f. YouTube
    g. Slack
    h. Discord
    i. Other
6. On average over the last two weeks, how much time is spent per day on social media platforms? This includes scrolling, posting, sharing, responding to comments, etc.
    a. Less than 1 hour
    b. 1-2 hours
    c. 2-4 hours

    d. 4-8 hours
    e. 8+ hours
7. What are your goals for using social media?
    a. Connect with friends
    b. Meet new people
    c. Keep up with trends
    d. Follow news and world events
    e. Pass time/avoid doing other things
    f. Distract myself
    g. Feel better emotionally
8. Social media is:
    a. Harmful
    b. Somewhat harmful
    c. Somewhat helpful
    d. Helpful
9. After viewing social media, I feel:
    a. Much worse about myself
    b. Somewhat worse about myself
    c. Somewhat better about myself
    d. Much better about myself
10. How does social media impact your mental health?
    a. Very positive
    b. Somewhat positive
    c. Somewhat negative
    d. Very negative
11. Are you in therapy or counseling for a mental health condition?
12. Are you currently taking medication for mental health?
13. Do you see content from anyone on social media who has or is recovering from an eating disorder?
14. If you have a history of an eating disorder, do you find eating disorder information on social media helpful or harmful?
15. What do you want adults to know about your time on social media?
